# A Gross Anatomy Ontology for Hymenoptera

**DOI:** 10.1371/journal.pone.0015991

**Published:** 2010-12-29

**Authors:** Matthew J. Yoder, István Mikó, Katja C. Seltmann, Matthew A. Bertone, Andrew R. Deans

**Affiliations:** Department of Entomology, North Carolina State University, Raleigh, North Carolina, United States of America; Field Museum of Natural History, United States of America

## Abstract

Hymenoptera is an extraordinarily diverse lineage, both in terms of species numbers and morphotypes, that includes sawflies, bees, wasps, and ants. These organisms serve critical roles as herbivores, predators, parasitoids, and pollinators, with several species functioning as models for agricultural, behavioral, and genomic research. The collective anatomical knowledge of these insects, however, has been described or referred to by labels derived from numerous, partially overlapping lexicons. The resulting corpus of information—millions of statements about hymenopteran phenotypes—remains inaccessible due to language discrepancies. The Hymenoptera Anatomy Ontology (HAO) was developed to surmount this challenge and to aid future communication related to hymenopteran anatomy. The HAO was built using newly developed interfaces within mx, a Web-based, open source software package, that enables collaborators to simultaneously contribute to an ontology. Over twenty people contributed to the development of this ontology by adding terms, *genus differentia*, references, images, relationships, and annotations. The database interface returns an Open Biomedical Ontology (OBO) formatted version of the ontology and includes mechanisms for extracting candidate data and for publishing a searchable ontology to the Web. The application tools are subject-agnostic and may be used by others initiating and developing ontologies. The present core HAO data constitute 2,111 concepts, 6,977 terms (labels for concepts), 3,152 relations, 4,361 *sensus* (links between terms, concepts, and references) and over 6,000 text and graphical annotations. The HAO is rooted with the Common Anatomy Reference Ontology (CARO), in order to facilitate interoperability with and future alignment to other anatomy ontologies, and is available through the OBO Foundry ontology repository and BioPortal. The HAO provides a foundation through which connections between genomic, evolutionary developmental biology, phylogenetic, taxonomic, and morphological research can be actualized. Inherent mechanisms for feedback and content delivery demonstrate the effectiveness of remote, collaborative ontology development and facilitate future refinement of the HAO.

## Introduction

Hymenoptera is an extraordinarily diverse lineage of insects, both in terms of species numbers and phenotypes, that includes sawflies (“Symphyta”), bees (Anthophila), wasps (numerous lineages), and ants (Formicidae). These insects serve critical ecological roles as herbivores, predators, parasitoids, and pollinators, with several species functioning as models for genomic (*e.g.*, *Apis mellifera*, the European honey bee) [1], behavioral (eusocial Aculeata, including the honey bee), virus coevolution [2], and evolutionary genetics (*e.g.*, *Nasonia* spp.) research [3].

**Figure 1:**
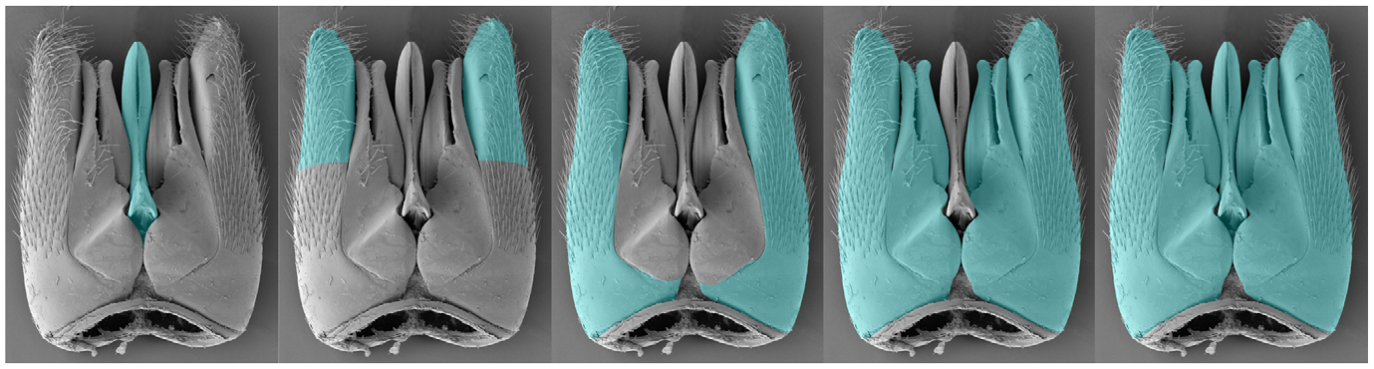
Usage of the term “paramere” as defined in different publications. The term “paramere” (highlighted in blue) is a homonym in this example, since it is used to describe different classes ( = concepts) in various publications. Within the HAO the A, B, C, D, E overlays respectively refer to the concepts HAO:0000707, HAO:0000395, HAO:0000389, (HAO:0000389+HAO: 0001084), and (HAO:0000707+ HAO:0000389+HAO: 0001084). See Introduction for citations.

The taxonomic and morphological diversity of Hymenoptera, combined with the vast array of researchers focusing on projects that span multiple domains, have yielded countless partially overlapping lexicons to refer to Hymenoptera anatomy. While we share common names for broadly understood structures, such as head, wing, leg, mandible, each community of specialized hymenopterists has its own terms that may or may not apply to shared structures across the phylogeny of this lineage. For example, the label “paramere” has been applied to at least five clearly incompatible sets of structures in the male genitalia (Fig. 1) [4]–[8]. The version of “paramere” used to make a statement about a given instance depends on the researcher making the observation and which of the hundreds of publications this individual is referencing as the source for anatomical terminology. Extreme cases of terminological disparity are found in anatomical systems that are under strong selection and/or have high value as a source of diagnostic characters for species. The external male genitalia (HAO:0000312) as a whole, for example, has been referred to by at least 13 English language terms. Disparate terminologies result in a vast sea of inconsistent but largely recoverable statements about hymenopterans. This situation inhibits future efforts to explore biological phenomena across Hymenoptera, including 1) comparisons of gene expression patterns, *e.g.*, mining genome annotations to understand the roles genes play in development, morphology and behavior [9]; 2) comparative morphology and phylogenetics, which is increasingly important as we attempt to include the vast amount of information from the fossil record and rare taxa from which we cannot extract DNA; 3) phenotype variability in the context of environment, *e.g.*, responses to variations in host quality, appropriateness, or nutrition in response to global climate change; 4) descriptive taxonomy and phenomics, as the corpus of descriptive taxonomy (each description is essentially a block of statements about a species' phenotype, often loosely following the Entity-Quality format used by annotators of genomic research [10]) cannot be efficiently queried for information to guide us on characters and species diagnosis; 5) computed reasoning, as logical, automated reasoning across our knowledge of hymenopterans requires a well-defined semantic framework.

Ontologies represent formalized domains of knowledge, whereby classes are related to one another to, in part, enable logical reasoning. For example, we have three classes: **leg**, **femur**, and **fore leg**, and these classes are related as: **femur** *part_of* **leg** and **fore leg** *is_a* **leg**. Given a statement, perhaps read in a species description, that “legs are yellow” we can reason that the fore leg must be yellow and that the femur must also be yellow since they each inherit this property from their parent class: leg. We are able to make inferences on the color of the femur and the fore leg—in the absence of explicit statements about the color of the femur and the fore leg—because we have an ontology that indirectly includes this information. That is, an ontology serves as a formal model through which one can employ mathematical logic to clarify and define concepts and relationships within a domain of interest. Like phylogenies, the tree-like patterns of historical relationships between organisms, ontologies link, and therefore provide critical contexts for biological concepts; in this way ontologies represent another mechanism through which inferences that inform our collective knowledge of evolutionary biology are generated. Ontologies make use of the transitive, symmetric, reflexive or other properties of the relationships between their concepts to derive logical conclusions from inexplicit information.

Given the incredible diversity of arthropods and the vast amount of associated information (especially with respect to genomics and evolutionary biology), there is a demonstrated need for controlled vocabularies that can link, unify, and clarify data between domains [11]. To date, there are only four other ontologies (Table 1) [12]–[15] that treat arthropod anatomy, none of which is as comprehensive with respect to taxa or intended scope. The *Drosophila* (FBbt), mosquito (TGMA) and tick (TADS) ontologies were developed as controlled vocabularies for literature and other annotations relevant to genomics [13], [14] and vector biology [12]. The spider ontology (SPD) [15], was developed to aid the organization of digital data (images) tied to anatomical concepts for use in phylogenetic analysis.

**Table 1:** A comparison of existing arthropod anatomy ontologies as reported from OBOEdit.

	Concepts( = OBO Terms)	# Terms Defined (% Total)	Relationship Distribution*	# Species covered**	Version			
			1	2	3	>3		
Hymenoptera (HAO)	1103	1103 (100%)	246	807	49	0	>150 k/1m	29:06:2010 13:39
Mosquito (TGMA) [12]	1861	1001 (92%)	1184	621	43	12	∼3500 (Culicidae)	04:02:2009 10:45
Tick (TADS) [12]	628	627 (99%)	380	189	46	12	∼900	18:11:2007 11:42
Drosophila (FBbt) [13], [14]	6570	1956 (30%)	2357	2968	813	431	1	04:02:2010 12:01
Spider (SPD) [15]	552	404 (73%)	342	197	10	1	∼40 k/∼150 k	17:03:2010 06:57

*Indicates the complexity of each ontology, with respect to the number of concepts with >1 or relationships.

**Estimate of presently described species/Estimated total world species (if notably different).

Through the Hymenoptera Anatomy Ontology (HAO) we seek to address terminological disparity by focusing and formalizing the efforts of anatomical experts and integrating their contributions with those of ontologists, computer scientists, and other domain experts. Deans and Ronquist (unpublished abstract from the 2006 International Congress of Hymenopterists, available through the HAO project website [16]) proposed a group effort to consolidate and relate the terminology we use for Hymenoptera anatomy. The initial objectives for this effort were to 1) converge on a common vocabulary that allows for easier comparisons across Hymenoptera and organize this vocabulary into a directed acyclic graph (DAG); 2) partner with Morphbank [17] to facilitate more efficient image searching using this DAG; 3) create an online glossary and browsable atlas of hymenopteran anatomy to serve as the “official” (*i.e.*, member-voted) resource for the International Society of Hymenopterists (ISH) and anyone interested in hymenopteran anatomy. The plan to develop a fully realized ontology, described herein, emerged from early work towards these initial goals, and we now intend the HAO to serve as a reference and learning tool and to enable efficient species description, data mining and retrieval, and literature annotation.

## Results

**Figure 2:**
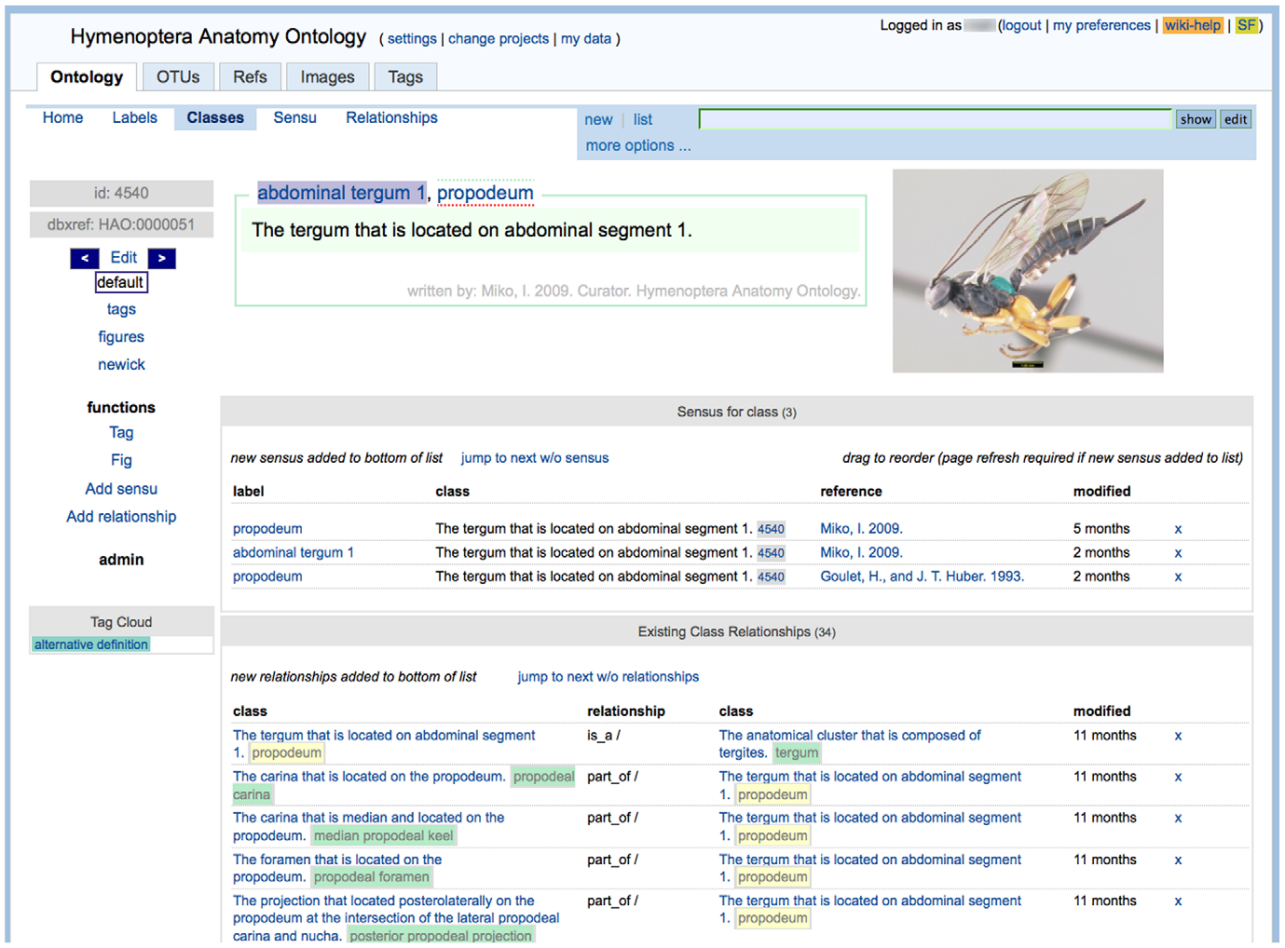
Screenshot of the curatorial interface for ontology classes. The class with labels “propodeum” and “abdominal tergum 1” is illustrated.

Multiple tools exist for the creation and management of ontologies [18]. Preliminary stages of ontology development, however, often involve disparate data gathering in the absence of an ontological framework. Some projects, including RDBOM [19], address this issue by providing Web-based platforms that are user-friendly to domain experts, who may not be adept at ontology construction. Mx [20], the open-source application in which the HAO was constructed and is now managed, embraces and facilitates this approach by providing a multi-feature environment for constructing ontologies. Key features include: 1) a Web-based, multi-user workspace; 2) *en mass* loading of terms via simple lists and simple text extraction mechanisms (*e.g.*, pasted-in blocks of text or statements stored within mx's database, including descriptive text or morphological character descriptions, can be parsed for candidate terms); 3) reference (and PDF) management, including EndNote import; 4) customizable annotation tools; 5) flexible search and browsing of the core ontological data (*e.g.*, Fig. 2); and 6) image management and annotation tools; Further information on this functionality and screenshots of mx's interface are documented on the mx wiki [20].

The database presently stores data from multiple ontologies, including our core data (Hymenoptera gross anatomy) and associated descriptive lexicons that are orthogonal to the HAO (*e.g.*, phenotypic qualities). The root class hierarchy is based on CARO [21].

**Figure 3:**
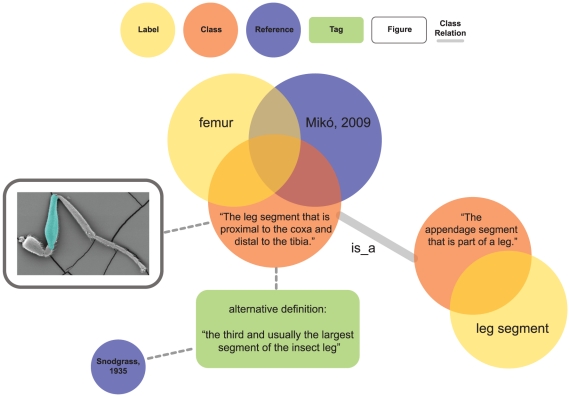
Simplified model of the data types in the HAO. Above- 6 of the 7 major data types, the 7th, a “sensu”, is described in Fig. 4. Below- an example with data from the HAO. A tag is a text annotation that references a keyword (from a configurable controlled vocabulary) and which may have additional notes, in this case the alternative definition. References can be associated in various places to provide additional context, as here in the relationship to a tag.

**Figure 4:**
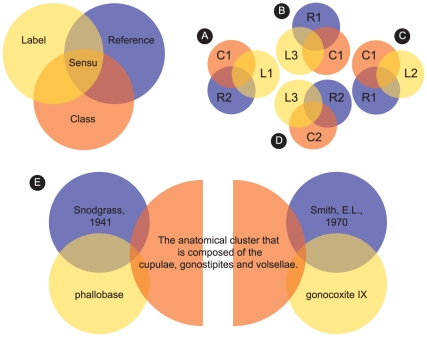
The *sensu* model. A *sensu* is the combination of a reference, concept and term. Given this model, independent observations (e.g. A–D) can be inferred across to compute synonyms (terms sharing classes), homonyms (a term with many classes), and acts of synonymy and homonymy. For example, given A–D, we can infer that: 1) T1, T2, and T3 are synonyms (all referenced C1); 2) T3 is homonymous (it refers to C1 and C2); and that 3) the author of R1 synonymized T2 and T3. An example from the HAO is provided in E. Here two sensus are defined both referencing the same class, the result being that the labels “phallobase” and “gonocoxite IX” are synonyms.

The HAO is the only arthropod anatomy ontology to provide all definitions as *genus-differentia* (see Methods) and is currently comprised of 2111 classes (1103 of which are defined and formally assigned HAO identifiers), 6977 labels (terms), and 3152 relations (properties) from *part_of*, *is_a*, and *attaches_to* (see Figs. 3, 4 for examples). There are 1772 text annotations on classes and 4711 on labels, *e.g.*, alternative definitions, indications of synonymy, indications of candidacy for external ontologies, tags facilitating internal workflow management, and personal annotations. Terms are tied to concepts with 4361 *sensus* (Fig. 4). Of the publications consulted, 177 are used in *sensu* records. There are 1073 images tied to classes as figures, and we recently initiated a process to annotate images with scalable vector graphic (SVG) overlays. Synonymy and homonymy among terms in the Hymenopteran literature are rampant. Even with our relatively small but directed sample size we have 1501 synonyms and 242 homonyms (∼50% and ∼8% respectively of terms tied to classes). Another outcome of this process is the accumulation of candidate terms for other ontologies, especially the Phenotype Quality Ontology (PATO) [22], for which we have 1134 candidates labels.

The HAO is a multispecies anatomy ontology whose structure and classes must be applicable across Hymenoptera (extinct and extant) and which will ultimately cover all multicellular anatomy exhibited by these insects. The HAO presently includes coverage for basic anatomical classes (mainly from CARO [21]): organism subdivisions, material and immaterial lines and surfaces, and integumentary modifications. The HAO is largely complete with respect to adult skeletal characters (sclerites, conjunctivae and integumentary modifications) and somatic muscles, which reflects the most referenced aspects of hymenopteran anatomy. Less well known, and therefore absent or minimally treated in the HAO, are the nervous system (brain, ganglia, nerves), the tracheal system, the circulatory system (pulsatory organs, heart muscles), exocrine glands, and the alimentary canal and corresponding accessory structures. Anatomy of immature stages (egg, larvae, pupae) are also not well-covered presently. A list of references we consulted is publically accessible through the Hymenoptera Glossary site [23].

## Discussion

The HAO is being developed as a foundational data source for all biologists referencing hymenopteran anatomy. Our goal is to facilitate the referencing of anatomical concepts across the biological sciences. Common references across domains will enable novel search, annotation, discovery and hypothesis testing. A shared resource is essential to the unified scientific exploration of the vast diversity of Hymenoptera. We anticipate that investigators examining morphological evolution and describing hymenopteran biodiversity will be the earliest adopters of this resource but that communities focused on model hymenopterans could easily implement the HAO using existing tools.

The HAO, excluding some candidate labels not presently tied to concepts, is accessible via the Hymenoptera Glossary site [23], which includes mechanisms for exploring the HAO: a search interface, a configurable tree-based browser, list reports, and an RSS feed that tracks changes throughout the core ontology. The OBO subset of the HAO, excluding some of the textual annotations used for internal maintenance, references to images, and candidate classes in the database, is available through the Ontology Lookup Service [24], [25] and BioPortal [26], [27]. Several other partnerships are underway, including the employment of the HAO to guide image searches within Morphbank, and the integration of the HAO with Ontobrowser, through Morphster.org [28]). Following the approach taken by Phenoscape [10], [29] the HAO is also being used within Phenex [30] to annotate new and previously published morphological matrices to build a corpus of phenotype statements.

Mechanisms for overlaying the HAO are also being developed. Text blocks can be vetted against the HAO and annotated [31], [32] with intersections translated to hyperlinks to class webpages. This utility, presently available as both a curatorial function and as a public “proofing” tool via the Hymenoptera Glossary [23], allows users to further explore the implied or potential meaning of underlying terms. Requests for changes, additions, or clarifications to the HAO can be posted to the HAO listserv (see HAO wiki for links [16]). A future version of the proofer tool will return URIs in a table format, allowing authors to footnote or append to their material and methods linkages between their concepts and the HAO. Digital publications will be able to directly link their descriptive text via hyperlinks to the provided URIs. This service will further be available as an API for external applications.

During the development of the HAO (and in the experiences of other anatomy ontology projects) the role of homology as a criterion for class definition and relations is frequently discussed, particularly when domain experts are introduced to the ontology. Though counterintuitive to some, the development of anatomy ontologies proceeds more effectively without reference to homology. Circumscriptions of classes in an ontology must first and foremost (at least within the goals of the HAO, and we feel in general) allow for the *identification of instances of the class in question*. These circumscriptions are crudely analogous to engineering blueprints in that they allow a domain expert to identify, with reference to an individual, some instance of a concept (*e.g.* the anterior ocellus on the specimen identified with the identifier NCSU 1234). Another central reason for decoupling anatomy from phylogeny (homology) is that it maximizes the potential cross-domain application of the ontology.

At their core, homology statements hypothesize that classes in two taxa are derived from a class in a third taxon (the common ancestor). These logical constructs are composed using ontologies that reference anatomy and taxonomy—for example, class B in some taxon Y *is_homologous_to* class C in some taxon Z *as* class A in taxon X—rather than being integrated within the anatomy ontology itself. There are, however, many similarities between the processes of generating accurate homology hypotheses and providing useful class circumscriptions within an anatomy ontology. A well-defined class will include characteristics that are often provided as criteria for determining homology (*e.g.*, topological correspondence or developmental origin). One could assume, therefore, that instances of a particular class in two individuals are by default homologous. The logical consequences of taking such a stance, however, are largely unexplored, and the HAO does not assume homology *a priori*.

The HAO inherently maintains a large number of taxonomies through its *is_a* and *part_of* hierarchies, for example “things that are part of the head.” Some morphological taxonomies are more controversial than others (*e.g*. systems of wing venation) and will undoubtedly require updating after more discussion and discovery. Circumscriptions that are determined to be unacceptable or otherwise ineffective can be changed, provided they remain logically consistent and well-defined. The HAO focuses on the clear delimitation of classes, rather than the nomenclature of labels applied to those classes, and therefore remains nimble with respect to adaptation and user preferences. A user can determine his/her preferred label and reference the associated class, with this term-concept coupling stored in the HAO as a *sensu* (see Materials and Methods and Fig. 4). While the present effort is focused on the English language, support for other languages is built in, and we encourage interested parties to work with us to provide multilingual support.

It has been stated that creating anatomical ontologies is “… just too complicated and difficult … to be enjoyable, and … no one in their right mind would even start making the anatomy ontology of an animal if they knew what they were getting themselves into!” [33]. Nevertheless it is clear that anatomical ontologies are poised to make transformative contributions in the scientific realm [34]. Existing difficulties are, in part, application based—*i.e.* ontology management is difficult, particularly to the domain experts with little or no experience in computer science, informatics, or philosophy. In developing new Web-based software that is available for cross domain construction of ontologies, and in testing and refining this software with a real-world ontology (HAO), we have contributed to easing the real (or, for the cynics, perceived) pain of creating anatomical ontologies.

The present state of the HAO, with respect to its relative completeness, unambiguous definitions, high level of documentation, and accessibility, illustrates the effectiveness of our approach to ontology development. Basic utility is already exemplified by several referencing approaches [31], [35], [36], and the HAO is accessible to machines (algorithms within OboEdit [37]), humans (“Hymenoptera Glossary” [23]), and both (e.g., BioPortal [26], [27]). We anticipate significant applications of the HAO, including a role in automated phenotype recognition and extraction [38], and utility in the annotation of taxonomic literature [39]. Those contributing to the HAO are, and will continue to be, acknowledged as members of the Hymenoptera Anatomy Ontology Consortium.

Future priorities for the HAO team are to 1) completely capture of the full anatomical lexicon for Hymenoptera (based on our sampling of optical character recognition (OCR)-processed text we estimate that 25% of the English Hymenoptera anatomy terms, most of which are obscure or are related to wing venation or internal morphology, remain to be incorporated into the HAO); 2) develop mechanisms (*e.g.*, visualizations, email surveys, user feedback interfaces, database APIs) by which stakeholders can participate in all aspects of the development and integration of the HAO; 3) begin to translate concept definitions into formalized semantic statements which are themselves part of the ontology; 4) illustrate all classes within the ontology; 5) develop mechanisms through which the core HAO data can be integrated into scientific papers referencing Hymenoptera anatomy (*e.g.*, taxonomic descriptions, phylogenetic analyses); and 6) align the HAO to other arthropod anatomy ontologies to exploit existing genomic and metabolic knowledge in those systems.

## Materials and Methods

**Figure 5:**
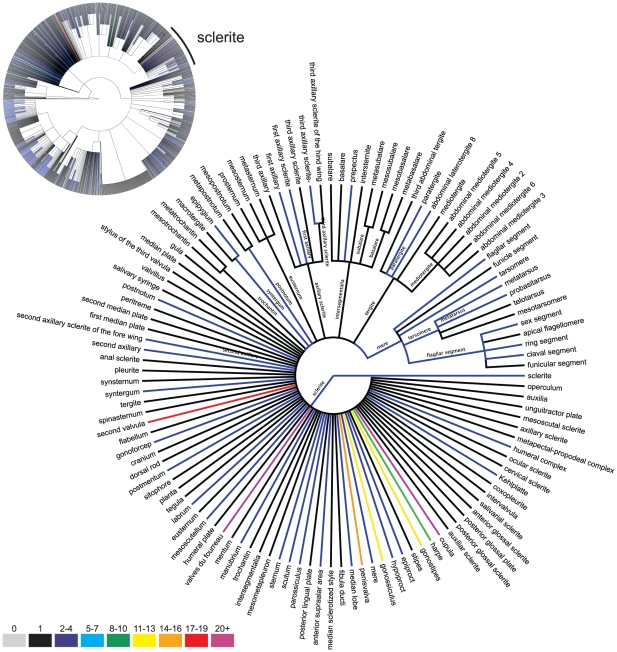
Illustration of the hierarchy annotation and visualization functionality of the ontology management features of mx. Top left: *is_a* hierarchy for all of HAO. Detail: sclerite hierarchy. Colors indicate the number of unique labels for the given class (edge). Tree exported from mx and drawn with Figtree (http://tree.bio.ed.ac.uk/software/figtree/).

The ontology-specific components of the application (Fig. 3) include separate models for classes ( =  concepts), labels ( =  terms), class relationship types (*e.g.* is_a, part_of), class relationships (*e.g.* the record that links two concepts via a class relationship: “concept B is_a concept A”), and *sensus*. Classes necessarily include a *genus-differentia* definition [40] and optimally include textual and visual annotations that clarify the definition. The application allows for classes to be generated with only these data, when the concept is deemed suitably well-documented, has been associated with a label via a *sensu* (see below), and given at least one *is_a* relationship to another class. The class is then assigned an identifier. Labels to be used in OBO exports are indicated in a 1:1 relationship of a label to a class in the database (*i.e.* the OBO format is a subset of the application functionality in this regard). The *sensu* model (Fig. 4) links the usage of a label to that of a concept, in the context of a citation, and allows for the computation of synonymy and homonymy across labels. Individual *sensu* records can be arbitrarily ordered with respect to classes to indicate a preferred label. Labels are language independent and have a one-to-many relationship with classes. An additional annotation layer global to the whole application allows tags and figures to be applied to all models. This layer allows for commenting, subsetting, and workflow-level annotations, or any other grouping functionality. Tags can indicate equivalences and/or candidacy in other ontologies (*e.g.*, cross-references or “xrefs”), alternative definitions, homonymy and/or subsets, and may be related to individual or team work-flow (*e.g.*, “quarantined until next revision,” “questionable definition,” or “needs review”). The ontological hierarchy can be visualized via a customized interface that exports data to a Newick format tree, which can be subsequently observed in Figtree [41] or other Newick tree visualizers (Fig. 5). Mx also includes functionality for mapping properties to the hierarchy, for example color-highlighting branches by metadata tied to references or the hierarchy itself (Fig. 5). The basic data model is format-agnostic, and multiple external formalizations of an ontology in mx can be created. The data described above are potential constituents in any formalization, and images and other metadata can be included as URI references.

Mx is a multi-user, multi-project Web-based application that handles a wide range of data, and the ontology is integrated with numerous internal functions. Within the database, blocks of text can be hyperlinked based on matches to the ontology, including descriptive text (*e.g*., morphological character descriptions, taxonomic descriptions, and identification tools like taxonomic keys).

The HAO integrates with Morphbank [17] using their Web services via a Ruby Gem created by MJY [42]. Images deposited in Morphbank are available to illustrate the ontology in mx under the Creative Commons BY-NC-SA license [43]. Included within Morphbank are ∼8800 images from the Hymenoptera Tree of Life (HymAToL) project. Many of the HymAToL images represent standard views, which are particularly useful for illustrative purposes.

Data were gathered for an initial period of three years from 962 references (the working list can be visualized on the Hymenoptera Glossary site [23]). Terms and, where applicable, their verbatim definitions were extracted from the literature. These data were then key stroked into the database, or terms were semi-automatically extracted from blocks of text via mark-up, extraction, and comparison to existing database content. Following the direct funding of the HAO project in early 2009 the process of formalizing existing data into an ontology according to the data model presented here was initiated.

Candidate OBO versions of the HAO are exported from mx and validated in OBO Edit [37] prior to being committed to the OBO Foundry repository [44]. “HAO” is used as a namespace/prefix for IDs of classes in the OBO file. The latest public release can be found in the OBO Foundry and is versioned with a time stamp. The OBO Foundry also provides a Web Ontology Language (OWL) formatted translation.

Additional details pertaining to all aspects of the HAO project are available at http://hymao.org. The HAO OBO file is available at http://www.obofoundry.org/cgi-bin/detail.cgi?id=hymenoptera_anatomy. The underlying software, ‘mx’ is written in Ruby, requires MySQL, and is available from SourceForge through links at http://purl.oclc.org/NET/mx-database. All software is open source. All exported data are available under a Attribution-NonCommercial-ShareAlike 3.0 Unported license.
